# Tuberculous coxitis with trochanteric bursitis manifesting a year after immigration to Germany: a case report

**DOI:** 10.1186/s13256-018-1867-9

**Published:** 2018-11-07

**Authors:** Georgios Sogkas, Anna Holz, Elke Riechers, Florian Länger, Christian von Falck, Reinhold Ernst Schmidt, Torsten Witte

**Affiliations:** 1 Hannover Medical University, Clinic for Immunology and Rheumatology, Carl-Neubergstr. 1, 30625 Hannover, Germany; 2 Institute for Pathology, Hannover Medical University, Carl-Neubergstr. 1, 30625 Hannover, Germany; 3 Institute for Diagnostic and Interventional Radiology, Hannover Medical University, Carl-Neubergstr. 1, 30625 Hannover, Germany

**Keywords:** Tuberculosis, Trochanteric bursitis, Coxitis

## Abstract

**Background:**

Osteoarticular tuberculosis is rare in Germany. In particular, trochanteric bursitis is an extremely rare manifestation of osteoarticular tuberculosis. We describe a case of tuberculous coxitis with trochanteric bursitis, successfully treated with a fourfold tuberculostatic therapy*.*

**Case presentation:**

We report the case of a 43-year-old human immunodeficiency virus-negative Sudanese man with osteoarticular tuberculosis, who was originally admitted with the suspected diagnosis of ankylosing spondylitis. Low grade fever together with the positive result of an interferon-gamma release assay test as well as findings from magnetic resonance imaging provided clues to the diagnosis. A definitive diagnosis could be set after a computed tomography-guided biopsy.

**Conclusions:**

Apart from a rare involvement pattern of osteoarticular tuberculosis, including trochanteric bursitis, this case highlights the increasing importance of osteoarticular tuberculosis as a differential diagnosis of rheumatic disorders. With the growing migration flows from tuberculosis-endemic African countries, clinicians in central and northern Europe may be more frequently confronted with atypical involvement patterns of osteoarticular tuberculosis.

## Background

Extrapulmonary tuberculosis may affect various organs, causing a broad range of clinical manifestations [1–3]. It provides an important source of mortality, especially if not diagnosed in time. Moreover, in cases of osteoarticular tuberculosis, misdiagnosis of an autoinflammatory disease and the consequent immunosuppressive therapy can further enhance morbidity and mortality of tuberculosis. Here we report the case of a 43-year-old African refugee, who was referred to our clinic with the suspected diagnosis of ankylosing spondylitis.

## Case presentation

**Fig. 1:**
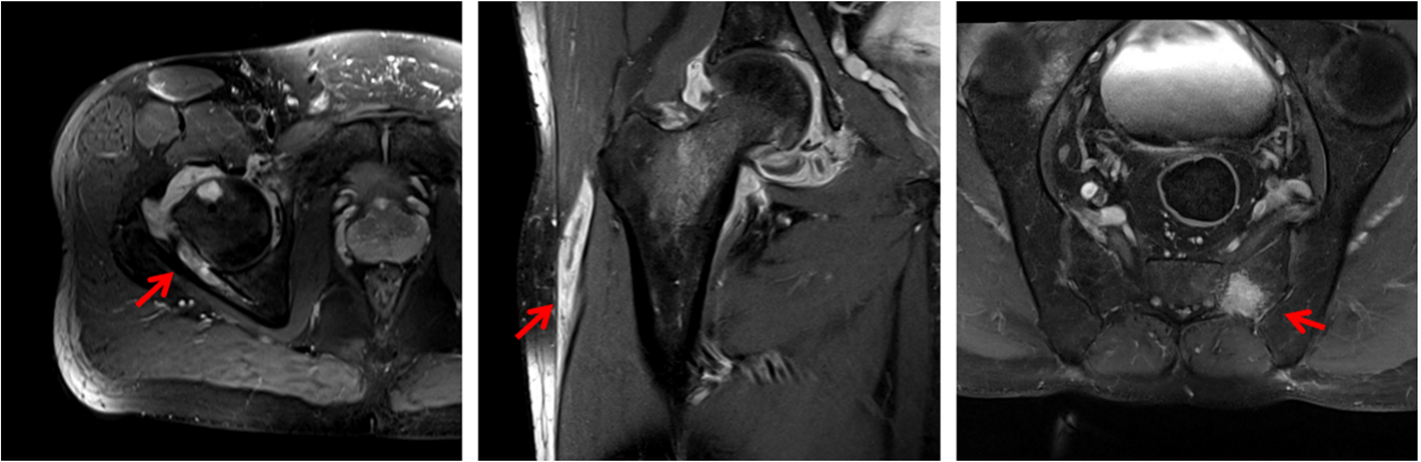
Magnetic resonance imaging scans; T2-weighted images. A right-sided coxitis (*right*) with a trochanteric bursitis (*middle picture*) as well as a left-sided osteitis of the sacral bone (*left*) are indicated with *red arrows*

**Fig. 2:**
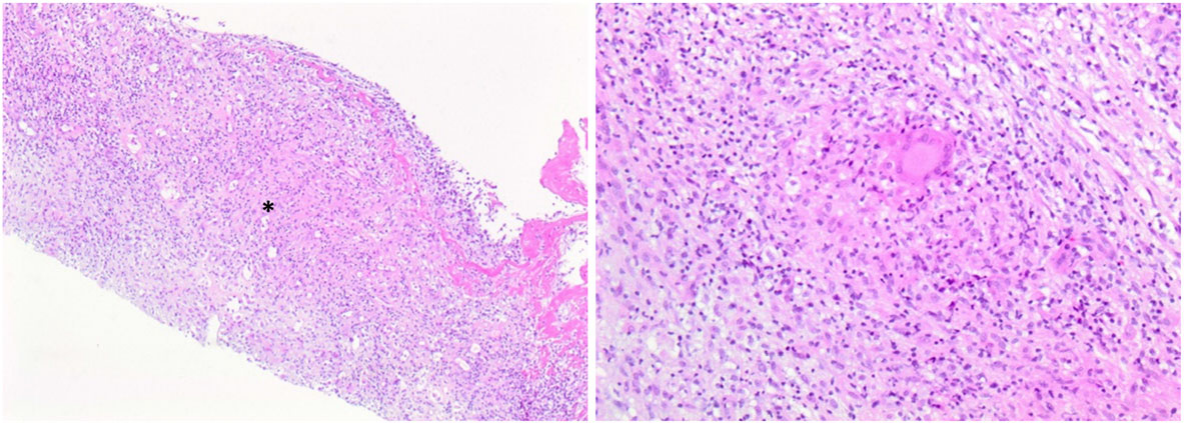
Histopathological examination: granuloma formation with caseous necrosis and epithelioid macrophages (left, 10× magnification; right, 40× magnification of the area, marked with a *star* on the *left-sided* photograph)

A 43-year-old man – a refugee Sudanese man – who had been living in Germany for 19 months was referred to our clinic by his rheumatologist with low back pain and the suspected diagnosis of ankylosing spondylitis. On admission our patient complained of low back pain radiating into his right hip, which started gradually during the last 4 months. He described the pain as persistent and more intense at night so that he could not sleep. Fever, weight loss, and night sweats were denied. In the clinical examination, movements of his right hip were painful. Before admission treatment with 25 mg prednisolone and diclofenac had been already initiated since spondyloarthritis was suspected. Prednisolone therapy was started 2 months before admission with an initial dose of 40 mg/day and was tapered to 25 mg/day until admission. On admission his erythrocyte sedimentation rate was normal and his C-reactive protein (CRP) was elevated (87 mg/l, normal range < 5 mg/l). The rest of routine laboratory tests were normal, except for a slight increase in gamma glutamyltransferase (gamma GT, 78 U/l; normal range < 55 U/l). The immunological tests showed a low titer of antinuclear antibodies (1:160) with no specification in the extractable nuclear antigen (ENA) screening test. The tests for rheumatoid factor and anti-citrullinated protein antibodies were negative. An HIV (human immunodeficiency virus) test was also negative. A conventional chest X-ray revealed no pathological findings. During his in-patient stay low grade fever was discovered. Conventional radiographs of his right hip, pelvis, sacroiliac joints, and his lumbar spine revealed osteosclerosis of his left sacroiliac joint without further abnormalities. In correlation with the pain in the area of his right hip, a pelvis and hip magnetic resonance imaging (MRI) scan revealed synovitis of his right hip with trochanteric bursitis (Fig. 1). Furthermore, there was left-sided osteitis of the sacral bone. We then performed an interferon-gamma release assay (IGRA), which was positive. Therefore, we collected material for microbiological as well as pathological diagnostics by puncture of his left hip joint. Histological examination of the periarticular tissue revealed the formation of granulomas with caseous necrosis (Fig. 2). Despite a negative acid-fast stain of the gained tissue sample, *Mycobacterium tuberculosis* was identified by polymerase chain reaction (PCR). Culture for mycobacteria remained negative. All other specimens submitted for microbiological testing (blood culture and sputum probes) remained culturally and PCR negative for *Mycobacterium tuberculosis.*

Based on the above described findings, we made a diagnosis of tuberculous coxitis with trochanteric bursitis. Molecular testing yielded no evidence of mycobacterial resistance to isoniazid or rifampicin. We therefore started a four-drug anti-mycobacterial regimen with rifampicin, isoniazid, ethambutol, and pyrazinamide. Prednisolone was gradually reduced until its complete discontinuation. After 2 months of therapy, ethambutol and pyrazinamide were stopped. Rifampicin and isoniazid were continued for 7 more months. In the follow-up, 6 months after starting therapy and 2 months after completion of a 9-month anti-mycobacterial regimen, his CRP was normal, and low back pain and painful motility of his right hip resolved completely. Conventional radiographs of his right hip showed a slight joint space narrowing with no further pathological findings. In the absence of functional limitation no surgical treatment was required.

## Discussion

According to the last “Global tuberculosis report” of the World Health Organization, the number of new cases of tuberculosis has tended to decrease over the last decades [1, 2]. Despite that, tuberculosis remains an important source of mortality, especially in developing countries. In 2014 tuberculosis caused 0.97–1.3 million deaths worldwide. In Germany, tuberculosis is relatively rare. However, in view of the recent developments in migration of refugees from Africa and Arabic countries to Europe, tuberculosis may become a more frequent entity in central and northern Europe in the future [4]. At present, osteoarticular tuberculosis accounts for 1–4.3% of all cases of tuberculosis [3]. In Germany, approximately 20% of newly diagnosed cases of tuberculosis had an extrapulmonary focus [5]. Osteoarticular tuberculosis accounts for 8.1% of newly diagnosed cases and is more common among patients of Asian or African origin. Tuberculous coxitis is thought to arise as a consequence of prior pulmonary tuberculosis through hematogenous spread of mycobacteria [3, 6]. Retrograde lymphatic or contiguous dissemination are also possible but less common.

Symptom onset is typically insidious (for example low grade fever, night sweats, and weakness) in cases of skeletal tuberculosis. Localized skeletal pain may appear later delaying the diagnosis of skeletal tuberculosis for many months [7]. The mean time until diagnosis after symptom onset has been reported to be approximately 16–19 months.

A positive IGRA or tuberculin skin test (TST) is crucial for diagnosing tuberculosis [8]. IGRAs have a higher specificity and sensitivity in comparison to TST. Despite the high specificity of the currently commercially available IGRAs, their sensitivity remains relatively low (ranging from 80 to 88% depending on the study and the assay) [9–11]. Skeletal tuberculosis presents no specific radiological findings [7, 12]. In cases of spinal tuberculosis, destruction of the intervertebral disc and both adjacent vertebral bodies is characteristic. The appearance of an osteolytic lesion can mimic metastatic tumors or primary osseous lesions. Computed tomography (CT) scans cannot discriminate between inflammatory and neoplastic lesions. MRI scans are more sensitive in showing early inflammatory lesions and, thus, suggesting the diagnosis of skeletal tuberculosis at an earlier stage. Typically, hypointense or isointense signals appear on T1-weigted images and hyperintense abnormalities appear on T2-weighted images of osseous and soft tissue lesions. The sensitivity of MRI can reach 100% whereas the sensitivity of CT may be around 88%.

A definitive diagnosis of extrapulmonary tuberculosis requires the obtaining of specimens from the relevant sites for microscopy and culture, although the sensitivity of these procedures is relatively low [13]. CT-guided or ultrasound-guided needle aspiration is essential for accurate diagnosis of skeletal tuberculosis [14], which is also reflected in the presented case. Revelation of granulomas and detection of mycobacteria by means of imaging-guided needle aspiration is the gold standard for diagnosing tuberculosis. Isolation of mycobacteria may have therapeutical consequences by revealing possible drug resistances. The application of sensitive molecular techniques can be extremely helpful especially in cases of samples with low numbers of mycobacteria.

## Conclusions

This case presents a rare involvement pattern of osteoarticular tuberculosis, including trochanteric bursitis. With the increasing migration flows from African countries, which are endemic for tuberculosis, osteoarticular tuberculosis arises as an important differential diagnosis of rheumatic disorders in central and northern Europe.

## References

[1] Lozano R, Naghavi M, Foreman K, *et al.* Global and regional mortality from 235 causes of death for 20 age groups in 1990 and 2010: a systemic analysis for the Global Burden of Disease Study 2010. Lancet. 2012;380:2095–128. 10.1016/S0140-6736(12)61728-0 PMC10790329 23245604

[2] World Health Organization. Global tuberculosis report 2015. Available at: http://www.who.int/tb/publications/global_report/en/. Accessed May 20, 2016

[3] N A, Ahmad F, Huda N. Osteoarticular tuberculosis-a three years' retrospective study. J Clin Diagn Res. 2013;7:2189–92. 10.7860/JCDR/2013/6859.3467 PMC3843457 24298472

[4] Jablonka A, Dopfer C, Happle C, Sogkas G, Ernst D, Atschekzei F, Hirsch S, Schäll A, Jirmo A, Solbach P, Schmidt RE, Behrens GMN, Wetzke M. Tuberculosis Specific Interferon-Gamma Production in a Current Refugee Cohort in Western Europe. Int J Environ Res Public Health. 2018;15(6):1263. 10.3390/ijerph15061263 PMC6025316 29904012

[5] Forssbohm M, Zwahlen M, Loddenkemper R, Rieder HL. Demographic characteristics of patients with extrapulmonary tuberculosis in Germany. Eur Respir J. 2008;31:99–105. 10.1183/09031936.00020607 17804450

[6] Enache SD, Pleasea IE, Anusca D, Zaharia B, Pop OT. Osteoarticular tuberculosis-a ten years case review. Rom. J Morphol Embryol. 2005;46:67–72. 16286988

[7] Moore SL, Rafii M. Imaging of musculoskeletal and spinal tuberculosis. Radiol Clin N Am. 2001;39:329–42. 10.1016/s0033-8389(05)70280-3 11316362

[8] Feng Y, Diao N, Shao L, Wu J, Zhang S, Jin J, Wang F, Weng X, Zhang Y, Zhang W. Interferon-gamma release assay performance in pulmonary and extrapulmonary tuberculosis. PLoS One. 2012;7:e32652. 10.1371/journal.pone.0032652 PMC3302779 22427859

[9] Diel R, Loddenkemper R, Nienhaus A. Evidence-based comparison of commercial interferon-gamma release assays for detecting active TB: a metaanalysis. Chest. 2010;137:952–68. 10.1378/chest.09-2350 20022968

[10] Sester M, Sotgiu G, Lange C, Giehl C, Girardi E, Migliori GB, Bossink A, Dheda K, Diel R, Dominguez J, Lipman M, Nemeth J, Ravn P, Winkler S, Huitric E, Sandgren A, Manissero D. Interferon-γ release assays for the diagnosis of active tuberculosis: a systematic review and meta-analysis. Eur Respir J. 2011;37:100–11. 10.1183/09031936.00114810 20847080

[11] Georgios S, Carl-Christoph P, Vivien S, Ludwig S, Ernst SR, Matthias S. A Case of New Manifestation of Leprosy Six Months after Immigration to Germany. Ann Clin Case Rep. 2017;2:1358.

[12] De Backer AI, Mortelé KJ, Vanhoenacker FM, Parizel PM. Imaging of extraspinal musculoskeletal tuberculosis. Eur J Radiol. 2006;57:119–30. 10.1016/j.ejrad.2005.07.005 16139465

[13] McNerney R, Cunningham J, Hepple P, Zumla A. New tuberculosis diagnostics and rollout. Int J Infect Dis. 2015;32:81–6. 10.1016/j.ijid.2015.01.012 25809761

[14] Jambhekar NA, Kulkarni SP, Madur BP, Agarwal S, Rajan MG. Application of the polymerase chain reaction on formalin-fixed, paraffin-embedded tissue in the recognition of tuberculous osteomyelitis. J Bone Joint Surg Br. 2006;88:1097–101. 10.1302/0301-620X.88B8.17625 16877614

